# The Role of EBV-Induced Hypermethylation in Gastric Cancer Tumorigenesis

**DOI:** 10.3390/v12111222

**Published:** 2020-10-28

**Authors:** Lyla J. Stanland, Micah A. Luftig

**Affiliations:** Center for Virology, Department of Molecular Genetics and Microbiology, School of Medicine, Duke University, Durham, NC 27710, USA; lyla.stanland@duke.edu

**Keywords:** Epstein–Barr virus, EBV, hypermethylation, CpG island, gastric cancer, tumor suppressor gene, differentiation

## Abstract

Epstein–Barr-virus-associated Gastric Cancer (EBVaGC) comprises approximately 10% of global gastric cancers and is known to be the most hypermethylated of all tumor types. EBV infection has been shown to directly induce the hypermethylation of both the host and viral genome following initial infection of gastric epithelial cells. Many studies have been completed in an attempt to identify genes that frequently become hypermethylated and therefore significant pathways that become silenced to promote tumorigenesis. It is clear that EBV-induced hypermethylation silences key tumor suppressor genes, cell cycle genes and cellular differentiation factors to promote a highly proliferative and poorly differentiated cell population. EBV infection has been shown to induce methylation in additional malignancies including Nasopharyngeal Carcinoma and Burkitt’s Lymphoma though not to the same level as in EBVaGC. Lastly, some genes silenced in EBVaGC are common to other heavily methylated tumors such as colorectal and breast tumors; however, some genes are unique to EBVaGC and can provide insights into the major pathways involved in tumorigenesis.

## 1. Introduction

Epstein–Barr Virus (EBV) is a ubiquitous human herpesvirus that transforms B lymphocytes and establishes lifelong latent infection. While infection is typically asymptomatic, in some patients, EBV is the causal agent of B cell and epithelial cell malignancies, including Burkitt’s Lymphoma, nasopharyngeal carcinoma (NPC) and gastric cancer [1]. In 1990, EBV was first detected via polymerase chain reaction (PCR) in gastric tumors and it is now known that approximately 10% of gastric cancers worldwide are EBV-associated [2]. EBV-associated gastric tumors (EBVaGC) are made up of monoclonal populations of EBV-infected epithelial cells suggesting that infection is an early event in tumorigenesis and a key oncogenic driver [3]. Shortly after the initial identification of EBVaGC, several groups set out to characterize the differences between infected and uninfected tumors and identified aberrant methylation patterns of cancer associated genes in the infected tumors. Most of this early work was done using techniques such as methylation specific PCR and pyrosequencing to probe single genes of interest [4,5,6]. However, more recently, several groups have utilized advanced whole methylome sequencing techniques to query methylation and hydroxymethylation status at the genome-wide level [7,8,9,10,11,12,13,14,15,16,17]. For example, The Cancer Genome Atlas Network (TCGA) completed a largescale, comprehensive analysis of 33 different tumor types, including gastric adenocarcinoma [9]. This study identified four distinct subtypes of gastric cancer: EBV infected, chromosomally instable (CIN), genomically stable (GS), and microsatellite instable (MSI). This study found that EBV-infected gastric tumors are molecularly, genetically and epigenetically distinct from the other three tumor subtypes. Whole methylome analysis of nearly 300 gastric tumors revealed that EBV-positive tumors display an extreme CpG-island hypermethylator phenotype (CIMP), resulting in hypermethylation of approximately 19% of promoter CpG-islands. The MSI subset of gastric tumors additionally show extreme CIMP, though these tumors are distinct from EBVaGCs both in the total quantity of methylated CpG-islands (10%) and the function of those genes. When compared to the other tumor types analyzed, such as colorectal (6%), and breast and glioblastoma (5%), it was found that EBV-positive gastric tumors are the most hypermethylated of all tumor types [9]. While many of the early studies of methylation of gastric cancer identified only of a handful of regulated genes, the TCGA study was a major step toward the characterization of the genome-wide methylome of these tumors.

In an effort to identify key pathways involved in tumorigenesis, and to better understand the importance of CIMP in EBV-positive tumors, several groups have more extensively characterized these tumors and developed in vitro models to study the role of methylation in tumorigenesis. As a result of these studies, we are gaining a clearer picture of the process of EBV infection, de novo methylation and tumorigenic cellular outgrowth, though much is still unclear. Whole methylome sequencing, pyrosequencing, and RNA sequencing have been completed in a number of cell lines, tumor tissue, and normal gastric tissue to identify EBV-specific patterns of methylation (Table 1). Some of the characterized cell lines include gastric cancer cell lines and SV40-transformed fetal gastric epithelial cells that have been infected with EBV in vitro [10,11,12,15,16,18,19,20].

Additionally, there are many gastric tumors, both EBV infected and uninfected, that have been sequenced and extensively characterized in an effort to identify EBV-specific epigenetic changes that occur following infection [3,9,14]. In the following sections of this review, we summarize the findings of the key studies of EBV-regulated methylation and other epigenetic changes that impact EBV-associated gastric cancer and how these relate to other EBV-positive and high CIMP tumor types.

## 2. EBV Infection of Gastric Epithelial Cells and Induction of Hypermethylation

**Figure 1:**
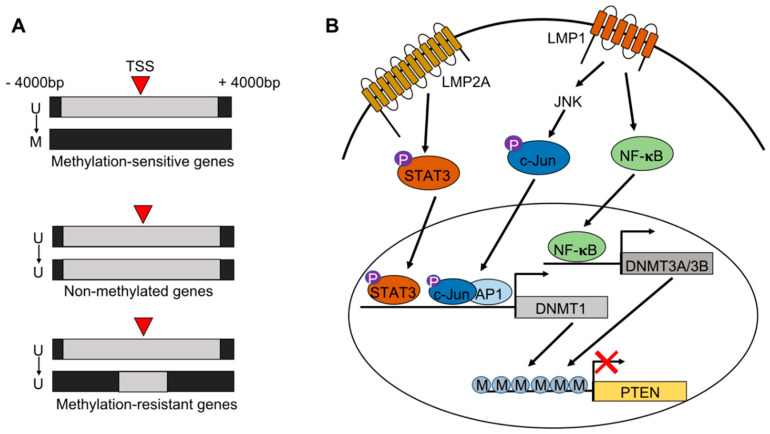
Methylation induction following Epstein-Barr virus (EBV) infection of epithelial cells. (**A**) Subtypes of genes that are unmethylated prior to EBV infection. Methylation occurs at and around the transcriptional start site (TSS). Genes can become methylated, remain unmethylated or appear to be methylation resistant, suggesting protection from silencing. (**B**) Pathways by which EBV latency proteins LMP1 and LMP2A can drive methylation. LMP2A can phosphorylate STAT3 which binds to the promoter region and activates transcription of *DNMT1*. Additionally, LMP1 can activate *DNMT1* through c-Jun/JNK signaling. LMP1 can also drive transcription of DNMT3A and DNMT3B through NFκB signaling. All DNMT proteins then methylate gene promoters, including TSGs such as *PTEN*.

EBV-associated gastric cancers arise following EBV infection and the transformation of gastric epithelial cells. It is thought that this typically occurs in patients with long-term gastric inflammation, dysplasia or chronic atrophic gastritis. One of the most significant processes that occurs during infection is the de novo hypermethylation of both the viral and host genomes. Multiple groups have shown that EBV infection of epithelial cells in vitro directly induces global non-random hypermethylation of the host genome [10,11,16,21]. More specifically, the Kaneda group has extensively characterized the spatiotemporal dynamics of EBV-induced hypermethylation in non-neoplastic cell lines [10]. Hypermethylation can occur either directly at the transcription start site (TSS) of a gene, or somewhere within a ±4000 bp region surrounding the TSS [10,16,20]. While methylation can occur surrounding the TSS, methylation within ±2000 bp of the TSS results in gene silencing [16]. The Kaneda group identified three distinct classes of genes based on their methylation status 28 days after initial infection. Genes that are unmethylated in wild type cells can be methylation resistant, methylation sensitive, or non-methylated following EBV infection, suggesting that some genes are somewhat protected from becoming methylated (Figure 1A) [10,20]. Genes that are considered methylation-resistant display increased CpG-island methylation surrounding the TSS except for a region within ±400 bp of the TSS allowing for continued gene transcription [20]. These genes are enriched for DNA repair genes, including *MSH2*, *MSH6*, and *MLH1*, a gene which is frequently mutated in the other subtypes of gastric cancer [20].

Methylation of the host genome begins within 8 days following initial infection. Acquisition of de novo methylation rapidly accelerates approximately two weeks after infection and subsequently decelerates by the third week of infection resulting in a week of high velocity methylation of the host genome [10]. Methyl groups added to the host genome are sustained long term and become vital for the survival of the cells. The Scott group used a transient infection model in lung carcinoma cell lines to study methylation after the loss of viral infection. Cell lines were infected with a recombinant EBV and passaged under selection pressure for 10 passages, and then selection pressure was removed and the viral episomes were lost. Analysis of the methylation patterns in the infected cells, and the cells that lost infection were similar, suggesting that EBV-induced epigenetic alterations are in fact long term and not dependent on viral infection status [21].

It has been shown that EBV-induced hypermethylation is in part driven by the EBV latency protein, latent membrane protein 2A (LMP2A). LMP2A is one of the few viral genes expressed in EBV-infected gastric epithelial cells and has been shown to both up-regulate expression of DNMT1, a key methyltransferase, and down-regulate ten-eleven translocation (TET) enzymes TET1 and TET2, which are key demethylating enzymes (Figure 1B) [11,22]. DNA methyltransferases (DNMTs) are responsible for adding a methyl group to the 5′ carbon position of a cytosine to make 5-methylcytosine (5-mc) leading to transcriptional silencing [23]. TET enzymes then convert 5-mc to 5′-hydroxymethylation (5-hmc) marks which can trigger further hydroxylation or allow for active demethylation by DNA repair machinery resulting in transcriptional activation. It has been shown in gastric cancer cells infected with EBV in vitro that the silencing of TET2 with a shRNA increased the total number of methylation-sensitive genes, thereby contributing to de novo methylation [11]. The EBV-mediated up-regulation of DNMT1 and down-regulation of TET enzymes can together promote the acquisition of 5-mc marks while preventing the transition to 5-hmc marks and thus silencing the transcription of key genes. Latently infected epithelial cells express very few EBV proteins because the viral genome additionally becomes heavily methylated and silenced by approximately 17 days after initial infection. However, RNA-sequencing from day 10 following initial infection shows that many viral latent and lytic genes are expressed, including another latent membrane protein, LMP1 [10]. LMP1 has been shown to activate DNMT1 through the JNK/AP-1 pathway, and DNMT3A and -3B through the NFκB signaling pathway in NPC cells (Figure 1B) [24,25]. Given that the acceleration of host genome methylation begins after day 10, it is possible that early after infection both LMP2A and LMP1 are expressed and function to activate the DNMTs and drive methylation prior to the methylation of the viral genome.

Methylation of the host genome occurs without clear bias to chromosome, though it is considered to be non-random. In addition to describing the spatiotemporal dynamics of methylation, the Kaneda group has defined distinct epigenotypes that show methylation of specific genes based on the overall level of CIMP within a tumor. The three distinct epigenotypes include: (i) genes that are specifically methylated in EBV-positive tumors, (ii) genes methylated in both EBV-positive and EBV-negative high CIMP tumors, and (iii) genes that are methylated in all gastric tumors, including the low CIMP tumors [19]. Genes often silenced in EBV-infected tumors include tumor suppressors, cellular differentiation genes, cell cycle genes, and genes involved in negative regulation of canonical oncogenic signaling pathways [4,5,7,10]. In addition to the silencing and down-regulation of these factors, EBVaGCs also display the up-regulation of pro-oncogenic factors [9,16,17].

## 3. Alternative Epigenetic Mechanisms of Gene Silencing

The methylation of host gene promoters is not the only epigenetic mechanism that can cause global host gene expression changes in EBV-infected gastric epithelial cells. Heterochromatin-associated histone modifications can additionally exert powerful control of host gene expression via alteration of promoters and enhancer regions [12]. Gene expression can be altered either through loss of activation marks, such as H3K27ac, or gain of repressive marks, such as H3K9me3 and H3K27me3 [12,20]. For example, the Kaneda group found that nearly 600 genes were significantly down-regulated in EBV-infected cells due only to repressive marks at enhancer regions, and not due to alterations at their promoters. Importantly, repressed enhancers were significantly associated with the silencing of TSGs, while activated enhancers were significantly associated with pro-oncogenic genes [12]. The EBV-induced alteration of histone methylation and acetylation marks has been characterized in other cell types, including B cells and nasopharyngeal epithelial cells, suggesting that this is a common mechanism for controlling global gene expression during infection [26,27,28]. Additionally, chromatin remodeling due to EBV latency proteins is quickly becoming a topic of interest as it is clear that this causes major alterations to gene expression of the host cell. In the following sections, we comprehensively review the data from studies describing both genes that have been silenced by direct promoter hypermethylation, and by alterations to histone modifications.

## 4. Tumor Suppressors

**Figure 2:**
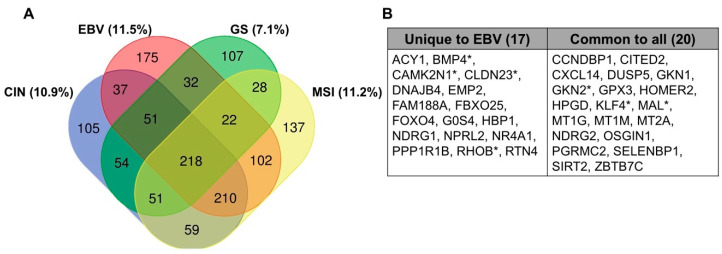
Down-regulated genes via mRNA-sequencing from the Cancer Genome Atlas dataset in gastric cancer subtypes. (**A**) Shown with percent of total genes that are known TSGs, identified from the TSGene Database. (**B**) TSGs listed that are unique to the EBV-infected subtype compared to those that are common to all subtypes. Asterisk indicates genes further described in Table 2 and Table 3.

It has been clear for several decades that EBV-induced hypermethylation of the host genome directly targets key tumor suppressor genes. The early studies of EBV-associated promoter methylation often utilized methylation specific PCR to focus specifically on known TSGs including *APC*, *PTEN*, *p14^ARF^*, *p15^INK4b^*, *p16^INK4a^*, *RASSF1*, *THBS1*, and *DAPK* [4,5,6,22]. With the emergence of improved genome-wide technologies to assay methylation status, the number of known silenced TSGs has grown and we are learning more about the pathways involved in tumorigenesis. Additionally, the TSGene Database has recently been updated and provides a comprehensive list of the 1217 known TSGs and their associated pathways that can be cross-referenced with existing datasets. For example, accessing data from the TCGA study of gastric adenocarcinoma shows that among genes down-regulated in each gastric cancer subtype, 11.5% are TSGs in EBVaGC, followed by MSI tumors (11.2%), CIN tumors (10.9%) and lastly GS tumors (7.1%, Figure 2A) [9]. There are TSGs that are commonly silenced across all four subtypes, and there are 17 that are unique to the EBV-infected subtype (Figure 2B).

Several of the silenced TSGs in EBVaGC are negative regulators of common oncogenic pathways including Wnt signaling, PI3K/AKT signaling and MAPK/ERK signaling. Specifically, this includes genes such as *RHOB*, *APC*, *SPINK5*, *SOX7*, *WNK2*, and *PTEN* (Table 2). Conversely, genes shown to be activated tend to be positive regulators of these pathways. Indeed, 80% of EBV-positive tumors additionally display activating mutations in *PIKCA* suggesting that these growth pathways are highly dysregulated [9,12,29,30]. For example, *TFF1* is found to be silenced in EBV-positive cells which can activate both NFκB signaling and β-catenin signaling pathways. TFF1 can also block the IL-6-mediated activation of STAT3 and is involved in normal gastric tissue health suggesting that loss of TFF1 will disrupt tissue integrity [31]. LMP2A has additionally been shown to promote cell survival through the up-regulation of survivin and activation of the NFκB signaling pathway, and activation of canonical Wnt signaling through β-catenin [29,32,33]. This suggests that, in combination, the silencing of key TSGs, plus the activation of pro-oncogenic factors by LMP2A, is important for the transformation and survival of EBV-infected gastric epithelial cells.

Several groups have defined a detailed mechanism for the direct promoter methylation and silencing of the key TSG *PTEN* that directly antagonizes the PI3K/AKT pathway. EBV latency proteins LMP2A and LMP1 have been shown to activate *DNMT1* through STAT3 signaling, and JNK AP-1 signaling, respectively, resulting in the methylation of the *PTEN* promoter [22,24]. LMP2A is only expressed in about 40% of EBVaGCs, while LMP1 is almost never expressed. However, as discussed in earlier sections, both mRNA transcripts were detected on day 10 after initial infection, suggesting that they may be expressed early and serve to turn on the transcription of DNMTs [10,22,25,34]. Indeed, LMP1 is a potent signaling molecule and little expression may be necessary to promote signaling. Direct mechanisms that may lead to the silencing of other TSGs have not yet been identified, though it is likely that EBV latency proteins play a major role similar to the mechanism of *PTEN* silencing.

Cell cycle genes are often dysregulated in cancer resulting in loss of normal checkpoints that act to prevent aberrant proliferation and oncogenic growth [35]. Many of the genes silenced in EBVaGC are classified as TSGs because of the roles that they play in cell cycle regulation. These genes include *THBS1*, *SFN*, *CAMK2N1*, *RHOB*, *RASSF1*, *APC*, *SFRP1*, *CDKN2A*, *CDKN1C*, *BMP4*, *BMP7*, *BRCA1*, *KLF4*, *OVOL1*, *TBX3*, and *RARA* [4,6,7,9,10,12,14,17,18]. While some of these genes are silenced in other tumor types as well, those that may be specific to EBVaGCs, including *RHOB*, *CAMK2N1* and *BMP4*. It is clear that EBV-induced hypermethylation of the host genome results in silencing of key TSGs and that increased genome-wide methylation results in the silencing of some factors that are typically not affected in other tumor types.

## 5. Differentiation Status

EBV-associated gastric cancers are typically diffuse-type tumors made up primarily of moderate to poorly differentiated epithelial cells with some lymphocytic infiltration [36]. Many of the genes silenced in EBVaGCs are cellular differentiation factors suggesting that EBV-induced hypermethylation can contribute to this phenotype (Table 3). Interestingly, EBV-positive NPC tumors are also made up of poorly differentiated cells suggesting that this is a conserved phenotype across EBV-associated epithelial tumors [1]. The early infection stage of gastric epithelial cells is poorly understood, and it is still unknown which cell types within the stomach are most likely to become latently infected. Given the diverse cell types present within the gastric crypt, the in vitro infection of cancer cell lines and monoclonal non-neoplastic cell lines does not accurately represent the environment in which EBVaGC occurs. However, the establishment of the latent infection of gastric tissue ex vivo has proven to be difficult and inefficient, and cell line models are still the most effective way to study EBV infection.

EBV is initially transmitted through saliva and infection of the host begins in the epithelial cells of the oral cavity. These are often highly differentiated epithelial cells that are thought to undergo lytic viral infection and produce large amounts of infectious virions [37,38]. Virions then enter the lymphoid tissue and infect naïve B cells [39]. Latent infection of these cells expands them, resulting in a pool of infected memory B cells that circulate in the blood throughout the entire life of the host [40].

**Figure 3:**
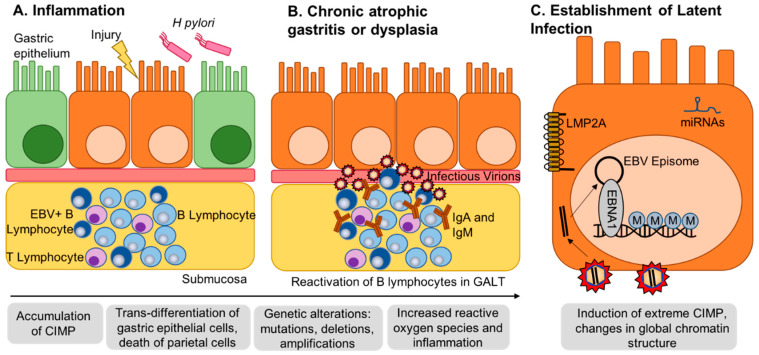
Gastritis-infection-cancer sequence of EBV-associated gastric cancer. (**A**) Local inflammation can be caused by numerous factors, including *H. pylori* infection or local injury. Prolonged inflammation results in chronic gastritis and the recruitment of lymphocytes to gut-associated lymphoid tissue (GALT) within the submucosa. (**B**) Chronic gastritis can progress into intestinal metaplasia or dysplasia characterized by the accumulation of CpG-island hypermethylator phenotype (CIMP) and the trans-differentiation of mature gastric epithelial cells. EBV-infected B lymphocytes reactivate to produce infectious virions with access to the basolateral surface of the epithelial cells. Normal B lymphocytes may additionally reactivate and produce IgA and IgM, which have been shown to mediate EBV attachment to epithelial cells. (**C**) EBV enters and establishes latent infection in epithelial cells. EBV latent proteins and miRNAs regulate methylation factors and induce de novo methylation in the host genome driving transformation.

**Figure 4:**
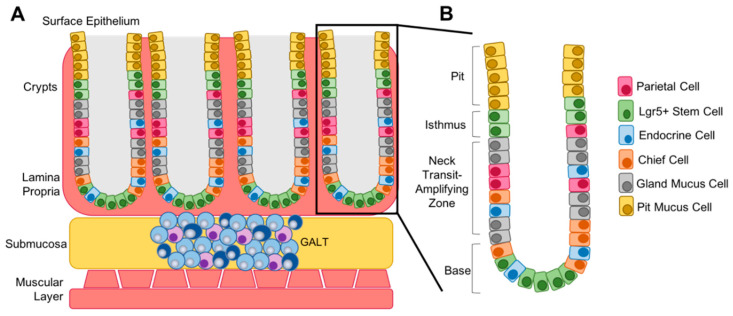
Structure of the gastric crypt. (**A**) In normal gastric tissue, crypts are located within the lamina propria, a layer of connective tissue, that lines the submucosa. The gut-associated lymphoid tissue (GALT) is located within the submucosa and is made up of T and B lymphocytes that reactivate upon injury, inflammation or infection. (**B**) The crypt is split into four main zones, the base that contains mostly Lgr5+ stem cells, the neck, which acts as the transit-amplifying zone, the isthmus, which contains additional stem cells, and the pit, which contains mature mucus cells that fill the villi that extend into the stomach.

B cells and T cells form secondary lymphoid tissues throughout the body, including localizing to the submucosa of the gut to form gut-associated lymphoid tissue (GALT, Figure 3). GALT is crucial to protect the body from infection via the gastrointestinal tract [41]. Inflammation, bacterial infection or local injury can lead to the activation of lymphocytes in the GALT. B cells that are latently infected with EBV will undergo viral lytic reactivation, resulting in the release of infectious virions to the basolateral surface of the gastric epithelium. It is thought that EBV primarily uses highly differentiated cells to undergo lytic replication and produce infectious virions, and that highly differentiated cells are unable to sustain latent infection as they are non-proliferative and often have become metabolically inert [15]. For this reason, it has been hypothesized that EBV infection in the stomach may occur primarily in poorly differentiated cell types within the base and isthmus of the gastric crypt. The base of the crypt typically contains Lgr5+ stem cells, while the isthmus is known as the transit-amplifying zone and contains both stem cells and proliferative progenitor cells that differentiate as necessary into mature cell types such as endocrine, parietal and chief cells to fill the crypt (Figure 4) [42,43].

The Mills Lab has found that gut epithelial cells display extreme plasticity and have the ability to both dedifferentiate and transdifferentiate through conserved molecular mechanisms [43,44]. Therefore, it is possible that virions can infect any cell type independent of differentiation status and induce hypermethylation of differentiation factors to revert cells to a less differentiated cell phenotype. Many of the genes shown to be silenced or down-regulated across multiple studies are positive regulators of cellular differentiation including *BMP4*, *BMP7*, *CDX2*, *FGF8*, *FGFR1*, *GATA5*, *MYADM*, *PROC*, *PROM1*, *RARA*, *SKIL*, *SMO*, *TGFB1*, *TGFB2*, *WNT7B*, *ZEB1*, *ZFP36L1* [7,9,10,12,14,15,17,18]. Gut stem cells are short lived and consistently proliferating making it difficult for them to accrue multiple mutations over time to fulfill the “multi-hit” hypothesis of carcinogenesis [44]. Mature cells, however, are long lived and can accrue mutations over time. It has been shown in NPC that the establishment of latent EBV infection requires a cell with existing genetic perturbations, termed a premalignant cell [45]. For example, the Tsao group showed that p16 loss and cyclin D1 over-expression resulted in successful latent infection of nasopharyngeal cells [45,46]. It is expected that a similar phenotype is required for the establishment of latent infection in gastric epithelial cells, which would support the hypothesis that infection occurs in mature cells with accrued mutations and hypermethylation drives dedifferentiation of the infected cells. In healthy gastric tissue, proliferation occurs mostly in poorly differentiated transit-amplifying cells; however, in chronic atrophic gastritis, mature cells such as parietal cells die and must be replaced by other cell types. It is thought that other mature cell types can re-enter the cell cycle and replace the cells lost [44]. The pathogenesis of EBVaGC is still somewhat unclear; however, it is possible that tumors develop in patients with gastritis, suggesting that, at the point of initial infection, mature cell types may be proliferating equally to stem cells and normal transit-amplifying cells.

Multiple groups have additionally shown that EBV infection of epithelial cells in vitro promotes anchorage independent growth, suggesting that cells are progressing through a process similar to the epithelial to mesenchymal transition (EMT) seen in vivo during oncogenesis [46,47,48,49]. Metastasis is common in gastric cancer patients and therefore it is expected that many genes silenced in EBVaGC are involved in the EMT. In a study completed at the M.D. Anderson Cancer Center, 66% of patients with EBVaGC displayed regional lymph node metastasis and EBV-infected metastatic tumor cells were found in the lymph nodes of all of these patients [3]. Some of the silenced genes in EBVaGCs include genes that are involved in maintaining planar cell polarity and cell-to-cell adhesion, such as *CLDN23*, *FBLN1*, *FUZ*, *VANGL1*, *VANGL2*, *CDH4*, *RHOB*, *MAL*, and *DVL*. Additionally, genes shown to be negative regulators of invasion and migration were silenced, including *CD109*, *CDH1*, *AHNAK* and *PLCD1* (Table 2 and Table 3, [7,9,10,12,14,18]. Dysregulation of these key pathways drives the EMT and results in the metastasis of tumor cells.

It is also likely that a poorly differentiated cell phenotype is better for the maintenance of latent infection and the prevention of lytic reactivation. Normalized oral keratinocytes (NOKs) infected with EBV appear to block differentiation induced by treatment with methylcellulose, while uninfected cells readily differentiated after treatment. Indeed, methylcellulose differentiation of NOKs promoted more changes in gene expression in uninfected cells compared to infected cells [15]. Additionally, several groups have found that EBV-infected epithelial cells grown in organotypic raft cultures are resistant to differentiation such that the suprabasal structure in the raft culture is disrupted in EBV-positive cells but properly structured in EBV-negative cells [15,50]. Cellular transcription factors known to be involved in epithelial cell differentiation, such as *ZEB1* and *KLF4*, can bind to viral promoters and activate the transcription of lytic genes such as *BZLF1* and *BRLF1* [15,50,51]. Viral gene expression and methylation of the viral genome will be discussed further in the following section.

## 6. Methylation of the Viral Genome

In addition to the host genome, the viral genome also becomes heavily methylated during the first month of infection to regulate both latent and lytic viral gene expression [10]. Methylation of the viral genome has been shown to occur more quickly than host genome methylation and is typically completed within 17 days after initial infection [10]. Latent and lytic genes can be detected by RNA-sequencing 10 days after infection, and, after this point, methylation accelerates and the expression of the majority of viral genes is lost. EBVaGC displays what is known as a latency I gene expression program where only a few key viral genes are expressed. This includes EBNA1, the non-coding RNAs known as EBERs, microRNAs from the *Bam*HI-A rightward transcript (BART) locus, and in approximately 40% of tumors, LMP2A [34,52]. Upon initial infection and entry into the cell, the viral genome becomes circularized and is maintained by the latency protein EBNA1, which tethers the EBV episome to the host chromosome. This process requires rearrangement of host chromatin to form structured interactions between the host and viral genomes [53,54]. In latently infected cells, the viral genome appears to have similar chromatin structure to the host chromosome. Additionally, a host cell chromatin binding factor, CTCF, binds the EBV genome in many locations and creates loops within the genome to facilitate long-distance enhancer and promoter interactions [54].

In infected cells that display the latency I gene expression program, EBNA1 is transcribed from the Q promoter (Qp), a constitutively active promoter that is typically hypomethylated in EBV-positive tumors [55]. However, three other viral gene promoters, Wp, Cp and Fp, become heavily methylated with the rest of the viral genome within 17 days of initial infection [10]. Methylation of the viral genome prevents expression of many viral proteins and this is thought to protect the virus from recognition by the immune system [56]. EBV-positive gastric tumors display high levels of lymphocyte infiltration, specifically CD8+ cytotoxic T cells and CD68+ macrophages, which could recognize surface viral antigens if they were expressed on the tumor cells [36].

It is clear that viral gene expression is controlled both by direct promoter methylation as well as alterations in active and repressive chromatin marks. Demethylation of the genome can be studied using drugs such as 5-Azacytidine, a cytidine analog and de-methylating agent, or histone deacetylase inhibitors, such as Romidepsin or Trichostatin A. Treatment of EBV-infected cells with these drugs leads to robust activation of transcription of viral lytic genes and production of infectious virions [21,53]. This can additionally result in the activated transcription of latent gene promoters Wp and Cp, and many host genes [6,21,57]. Epigenetic silencing of the viral genome plays a key role in transforming and maintaining latent infection in gastric epithelial cells, as well as other cell types that EBV infects in vivo.

## 7. Methylation in Other EBV-Infected Cell Lines and Tumors

EBV infection has been shown to induce methylation in the other cell types it is known to infect, namely, B cells and oral epithelial cells. Approximately 90% of Nasopharyngeal carcinomas are EBV-associated and while whole methylome studies of NPC have been slightly lacking compared to EBVaGC, interest in this area is increasing and we are learning more about the epigenetic landscape of NPC. It is now known that NPC is additionally a highly methylated tumor type due to EBV infection though methylation of NPC seems to be more strongly associated with chromosomal location. While EBV-induced hypermethylation in gastric epithelial cells is considered non-random, it does not appear to bias any specific chromosome. Conversely, EBV-induced hypermethylation of nasopharyngeal epithelial cells shows strong bias to chromosomes 3p, 6p and 9p, targeting many specific TSGs. However, a recent study found that both NPC and EBVaGC display a significant methylation peak at the chromosome region 6p21.3 [8]. This chromosomal region contains several genes that are known to be involved in oncogenesis as tumor suppressors, and the human leukocyte antigen (HLA) genes that are key determinants of risk in NPCs [8,58].

EBV is also causally associated with endemic Burkitt’s Lymphoma (eBL) and compared to uninfected B cells, BL tumor cells also show increased global methylation. Typically, eBLs show lower global mutation rates and are driven by an Ig/MYC translocation, indicating that epigenetic alterations may play a major role in these tumors as well [59,60]. Conversely to EBV-associated tumors, in vitro infected B cell lines known as lymphoblastoid cell lines (LCLs) are known to display regions of extreme hypomethylation when compared to normal uninfected cells. Many of the sites that are hypermethylated in the LCLs are conserved from normal B cells and are not a result of EBV-induced hypermethylation. The regions that become hypomethylated in LCLs are typically low CpG-island regions of the promoters near the TSS. This suggests that low CpG-island promoters that are typically methylated in normal B cells are sensitive to demethylation after infection with EBV, contrary to hypermethylation that occurs in epithelial cells [61]. Hypomethylation of genes is associated with the conversion of normal B cells to LCLs. However, regional methylation still occurs in LCLs and mainly targets TSGs, some of which are also targeted in EBVaGC such as *CDKN2A* (p14/p16), *TP73*, *DAPK*, *TGFB1*, and *CDKN2B* (p15) [4,5,6,10,12,14,18,26].

## 8. Comparison to Other Heavily Methylated Tumor Types

In normal tissue, approximately 1–2% of promoter CpG islands are methylated and often correspond with x-inactivation and germ cell specific expression patterns. In cancer tissues, the frequency of total promoter CpG islands that can be hypermethylated increases and results in gene silencing that promotes tumorigenesis [62]. EBV-positive gastric tumors display a genome-wide promoter hypermethylation frequency of approximately 19%. The next most hypermethylated tumors types are CIMP-high colorectal (13%), MSI-gastric (10%), CIMP-low colorectal (6%), and breast and glioblastoma (5%) [9]. There are genes that are commonly epigenetically silenced across the majority of tumor types, for example, *CDKN2A*, which encodes key cell cycle regulators p16 and p14. Conversely, there are genes that are commonly silenced in many other tumor types, and do not appear to be silenced in EBVaGC, such as *MLH1* and *MSH2* [9]. Gene ontology analysis of genes silenced in breast and colorectal tumors shows alterations in the Wnt signaling pathway, pathways involved in the regulation of differentiation status, and known tumor suppressors, similar to what is seen in EBVaGC [9]. EBVaGC tumors and high CIMP colorectal tumors both show common hypermethylation of *SFRP1*, which acts as a key regulator of Wnt signaling [9,10,63]. Additionally, high CIMP colorectal tumors specifically show similarity to EBVaGC tumors in the silencing of key polycomb complex associated genes involved in chromatin regulation [63]. High CIMP cases of glioblastoma also show strong co-occurrence with mutations in chromatin modification genes [64]. This suggests that the combination of hypermethylation and dysregulation of chromatin structure are key synergistic oncogenic pathways that occur in multiple different tumor types.

Interestingly, while 80% of EBVaGC tumors display an activating mutation in *PIK3CA,* high CIMP breast tumors are strongly enriched for low mutation rates of *PIK3CA*, *MAP2K4,* and *MAP3K1* [9,65]. High CIMP breast tumors are enriched for the luminal B subtype, which is known to be fast growing and display low survival rates [65]. This may suggest that the hypermethylation of key genes in high CIMP breast tumors can provide key pro-tumorigenic signals similar to what is seen in EBVaGC. CIMP status in colorectal tumors additionally shows enrichment for specific mutations as high CIMP tumors show high rates of *BRAF* mutation while low CIMP tumors show high rates of *KRAS* mutation [66]. Methylation does occur in other subtypes of gastric cancer as well. Compared to the other three gastric cancer subtypes, there are genes that are only hypermethylated in EBVaGC, including *CDH1*, *PTEN*, *RASSF1A*, *MGMT*, *MINT2*, *p15INK4B*, *p73*, *HOXA10*, *SSTR1*, *FHIT*, *CRPB1*, *WWOX*, *DLC1*, *HOXA11* (Figure 3) [9]. It is clear that there are both common, and unique pathways that are involved in EBV-associated gastric tumorigenesis compared to other gastric tumors as well as other high CIMP tumors.

## 9. Conclusions

It is evident that, even with the number of studies that have been completed, we do not have a clear picture of the factors that are most heavily involved in tumorigenesis after initial EBV infection. Many of the studies that have been completed have resulted in vastly different lists of genes that are detected as methylated or down regulated. This may be due to the differences in cell lines, sequencing techniques and analysis pipelines used. Even the total number genes identified as being epigenetically silenced can vary across data sets from as few as several hundred, to up to 7000 [11,13]. Additionally, much of what we currently know about EBVaGC has come from the characterization of tumors or the infection of already transformed cell types *in vitro*. In the future, it will be important to develop infection models in primary gastric cell types and tissue to study EBV-induced hypermethylation in non-transformed cells.

The specific pathogenesis of EBV-associated gastric cancer is additionally still unclear. It is thought that gastric epithelial cells without prior genetic perturbations are not capable of sustaining latent EBV infection and that a pre-malignant cellular phenotype is required. This suggests that patients with existing conditions such as chronic gastritis, dysplasia or intestinal metaplasia may be at risk for the development of EBVaGC. Prolonged inflammation of the gut can result in the accumulation of CIMP and it has been shown that high CIMP intestinal metaplasias display methylation of over 2500 genes, and 98.6% of those genes were also found to be methylated in EBVaGC [13]. Further analysis of these genes may shed light on pathway perturbations that precede EBV infection in comparison to pathway perturbations that occur following infection and virally induced methylation. It is imperative that we understand this process as this information can be utilized to screen patients with dysplasia or gastritis to identify patients that may be at the highest risk for the development of EBVaGC.

## Figures and Tables

**Table 1:** Details of the largescale datasets used in this review with data accession numbers where available. Gene lists for analysis were downloaded where available as supplemental files in original papers. First author and year listed with reference number.

Author	Dataset	Tissue	Array	Platform	Samples
Matsusaka 2011 [19]	GSE31789	Gastric	Methylation and Expression	GPL370 GPL8490	Gastric cell lines and primary tumors
Zhao 2013 [18]		Gastric	Methylation		Gastric cancer cell line ± EBV infection
Birdwell 2014 [16]	GSE59843	NOKs	HT Seq	GPL16791	Normalized oral keratinocytes
Bass 2014 [9]	TCGA Portal	Gastric	WGS, Methylation, RNA-seq	GPL8490 GPL13534	Gastric tumors and normal gastric tissues
Liang 2014 [7]	SRA67982	Gastric	WGS	GPL9115	Gastric cancer cell line ± EBV infection
Dai 2015 [8]	GSE62336	NPC	Methylation	GPL13534	Matched NPC and normal tissue from same donor
Namba-Fukuyo 2016 [11]	GSE84897	Gastric	RNA-seq, MeDIP-seq	GPL18460 GPL18573	Gastric cancer cell line ± EBV infection
Okabe 2017 [12]	GSE97837 GSE97838	Gastric	ChIP-Seq, FAIRE-seq	GPL10999 GPL18460	Gastric cancer cell line ± EBV infection
Huang 2017 [13]	GSE103186	IM	Methylation	GPL13534	Intestinal metaplasias and normal gastric tissue
Matsusaka 2017 [10]	GSE89269	Gastric	Methylation	GPL13534	Gastric cell lines, tumors, and normal gastric tissue
Liu 2018 [14]	GDC Legacy Archive	Gastric	Methylation	GPL8490 GPL13534	Gastrointestinal adenocarcinomas
Edwards 2019 [17]	PRJNA503182 PRJNA501807	Gastric and NPC	RNA-seq	GPL20301	Gastric and NPC cell lines ± EBV and xenograft
Eichelberg 2019 [15]	PRJNA555053	NOKs	RNA-seq	GPL18573	Normalized oral keratinocytes ± EBV infection, and methylcellulose

**Table 2:** TSGs silenced in EBVaGC. TSGs were identified using the TSGene Database of 1217 known TSGs. Lists of silenced and down-regulated genes from available data sets were cross-referenced and selected genes were present in multiple datasets as noted in the references column.

Gene	Location	Full Name	Function	References
*TGFBR3*	1p33–p32	Transforming growth factor beta receptor type 3	TGF-β signaling	[9,12]
*SFN*	1p36.11	Stratifin	Cell cycle checkpoint, p53 activator	[4,12]
*CAMK2N1*	1p36.12	Calcium/calmodulin dependent protein kinase II inhibitor 1	Cell cycle, apoptosis	[9,14]
*TP73*	1p36.3	Tumor protein p73	Apoptosis, DNA damage response	[10]
*GKN2*	2p13.3	Gastric motility protein 2	Proliferation, apoptosis, invasion	[9,12]
*RHOB*	2p24	Ras homolog gene family member B	Negative regulator of intracellular signaling, cell-to-cell adhesion	[9,12]
*MAL*	2q11.1	Myelin and lymphocyte protein	Integral membrane protein, anti-metastatic in epithelial cancers	[9,12]
*RASSF1A*	3p21.3	Ras association domain family member 1	Apoptosis, cell cycle checkpoint	[4]
*PLCD1*	3p22.3	Phospholipase C delta 1	Motility, migration and invasion, cytoskeleton reorganization	[9,14]
*APC*	5q21–q22	Adenomatous polyposis coli	Negative regulator of β-catenin, cell adhesion	[4]
*SPINK5*	5q32	Serine protease inhibitor Kazal-type 5	Inhibitor of Wnt signaling, anti-proliferation, migration and invasion	[12,17]
*AKAP12*	6q24–q25	A-kinase anchor protein 12	PKA scaffold protein, anti-metastatic	[10,12]
*DFNA5*	7p15	Deafness associated tumor suppressor	Apoptosis, regulated by p53	[10]
*SFRP1*	8p11.21	Secreted frizzled related protein 1	Growth inhibitory, anti-proliferative	[9,10]
*SOX7*	8p21.3	SRY-box transcription factor 7	Antagonism of Wnt signaling, anti-proliferation	[10]
*CLDN23*	8p23.1	Claudin 23	Cell polarity	[9,12]
*CDKN2A*	9p21	Cyclin dependent kinase inhibitor 2A	Cell cycle	[4,5,6,9,14,18]
*TUSC1*	9p21.2	Tumor suppressor candidate 1	Cell growth	[9,14]
*WNK2*	9q22.3	WNK lysine deficient protein kinase 2	Proliferation, negative regulator of ERK/MAPK pathway	[12,14]
*PTEN*	10q23.3	Phosphatase and tensin homolog	Negative regulator of PI3K/Akt signaling	[4,22]
*CDKN1C*	11p15.5	Cyclin dependent kinase inhibitor 1C	Cell cycle, anti-proliferation	[7,9,12]
*AHNAK*	11q12.2	AHNAK nucleoprotein	Invasion and migration, EMT	[9,12]
*BMP4*	14q22–q23	Bone morphogenetic protein 4	TGF-β signaling	[5,9,10,12]
*YPEL3*	16p11.2	Yippee-like 3	P53 inducible, anti-proliferation, cellular senescence	[9,12,14]
*FBLN1*	22q13.31	Fibulin 1	Cell morphology, growth, adhesion, motility	[9,10,12]

**Table 3:** Genes involved in cellular differentiation that are often silenced in EBVaGC. Differentiation genes were identified from the gene ontology term epithelial cell differentiation pathway (GO:0030855). Lists of silenced and down-regulated genes from available data sets were cross-referenced and selected genes were present in multiple datasets as noted in the references column.

Gene	Location	Full Name	Function	References
*VANGL1*	1p13.1	VANGL cell polarity protein 1	Planar cell polarity, columnar epithelial structure	[14]
*VANGL2*	1q23.2	VANGL cell polarity protein 2	Planar cell polarity, columnar epithelial structure	[9,10,14]
*DVL*	1p36.33	Disheveled	Cell adhesion, cell polarity	[12]
*CD109*	6q13	CD109 molecule	Negative regulator of EMT, invasion and migration	[10,12]
*MAP7*	6q23.3	Microtubule-associated protein 7	Microtubule structure, cell polarization	[12,14]
*CAV1*	7q31.1	Caveolin 1	Proliferation, migration, differentiation	[9,10]
*KLF4*	9q31	Kruppel-like factor 4	Proliferation, differentiation, apoptosis	[9]
*OVOL1*	11q13.1	Ovo like transcriptional repressor 1	Terminal differentiation, anti-proliferative	[9,14]
*TBX3*	12q23–24.1	T-box transcription factor 3	Transcription factor activates differentiation genes, cell cycle inhibition	[9,14]
*CDX2*	13q12.2	Caudal type homeobox 2	Pro-differentiation of intestinal epithelial cells	[9,10,14]
*SCEL*	13q22.3	Sciellin	Terminal differentiation	[12,17]
*CDH1*	16q22.1	E-cadherin	EMT, cell adhesion	[4,5,6,12]
*RARA*	17q21.2	Retinoic acid receptor alpha	Pro-differentiation and anti-proliferation	[7,12,18]
*FUZ*	19q13.33	Fuzzy planar cell protein	Planar cell polarity	[9,14]
*FOXA2*	20p11	Forkhead Box A2	Drives differentiation of mature gastric cell types	[7,12]
*BMP7*	20q13.31	Bone morphogenetic protein 7	Pro-differentiation and anti-inflammatory	[14,17,18]
*CDH4*	20q13.3	Cadherin 4	Cell adhesion, cell polarity	[10,18]
*GATA5*	20q13.33	GATA binding protein 5	Pro-differentiation	[7,17]
*TFF1*	21q22.3	Trefoil factor 1	Anti-inflammatory, pro-apoptotic, maintain gastric tissue health and integrity	[6,17]
